# Solving gap metabolites and blocked reactions in genome-scale models: application to the metabolic network of *Blattabacterium cuenoti*

**DOI:** 10.1186/1752-0509-7-114

**Published:** 2013-10-31

**Authors:** Miguel Ponce-de-León, Francisco Montero, Juli Peretó

**Affiliations:** 1 Departamento de Bioquímica y Biología Molecular I, Facultad de Ciencias Químicas, Universidad Complutense de Madrid, Ciudad Universitaria, Madrid 28045, Spain; 2 Departament de Bioquímica i Biologia Molecular and Institut Cavanilles de Biodiversitat i Biologia Evolutiva, Universitat de València, C/José Beltrán 2, Paterna 46980, Spain

## Abstract

**Background:**

Metabolic reconstruction is the computational-based process that aims to elucidate the network of metabolites interconnected through reactions catalyzed by activities assigned to one or more genes. Reconstructed models may contain inconsistencies that appear as gap metabolites and blocked reactions. Although automatic methods for solving this problem have been previously developed, there are many situations where manual curation is still needed.

**Results:**

We introduce a general definition of gap metabolite that allows its detection in a straightforward manner. Moreover, a method for the detection of Unconnected Modules, defined as isolated sets of blocked reactions connected through gap metabolites, is proposed. The method has been successfully applied to the curation of *iCG238,* the genome-scale metabolic model for the bacterium *Blattabacterium cuenoti*, obligate endosymbiont of cockroaches.

**Conclusion:**

We found the proposed approach to be a valuable tool for the curation of genome-scale metabolic models. The outcome of its application to the genome-scale model *B. cuenoti iCG238* is a more accurate model version named as *B. cuenoti iMP240.*

## Background

Metabolic reconstruction is the computational-based process that aims to elucidate the network of metabolites interconnected through reactions catalyzed by activities assigned to one or more genes [1-5]. The reconstruction process begins with the functionally annotated genome of an organism. Then, the identification of those genes whose putative products catalyze some biochemical reaction, *i.e*. gene products with an assigned enzyme commission number (EC) or transport commission number (TC), should be done. This relational information can be organized in the so-called gene-protein-reaction association tables (GPR) [1]. In a further step GPR tables will be used to infer candidate metabolic pathways coded in the organism’s genome [2].

In order to automate the process of reconstruction of a target organism’s metabolic network, computational methods have been previously developed that will yield a first draft [6-8]. This first draft can be used to formulate a mathematical representation of an organism’s metabolism, termed as genome-scale model (GSM). The Constraint-Based-Modeling (CBM) is an approach that combines the stoichiometric analysis with optimization techniques to study genome-scale models [9-14]. The CBM has been successfully used to predict metabolic capabilities such as growth rates, as well as systems responses to environmental or genetic perturbations [15-18].

When applying CBM to an initial draft of a metabolic model, it is usual to find inconsistencies that can have different causes. In the initial stages of a metabolic reconstruction, due to annotation errors, as well as the existences of unknown enzyme functionality, GPR associations can be incorrectly established. Thus, some reactions may be not included in the model draft. As a consequence, some metabolic pathways will contain gaps that will create dead-end metabolites [19]. These metabolites appear in the model as only produced or only consumed by reactions, and hence will never reach a steady state different than the trivial, and then they will never participate in a feasible solution. They will in turn block any reaction in which they are involved. There are two classes of dead-end metabolites: i) Root-Non-Produced metabolites (RNP) *i.e*. metabolites that are only consumed by system’s reactions, and ii) Root-Non-Consumed (RNC) that includes those metabolites that are only produced by the network but never consumed [20].

**Figure 1:**
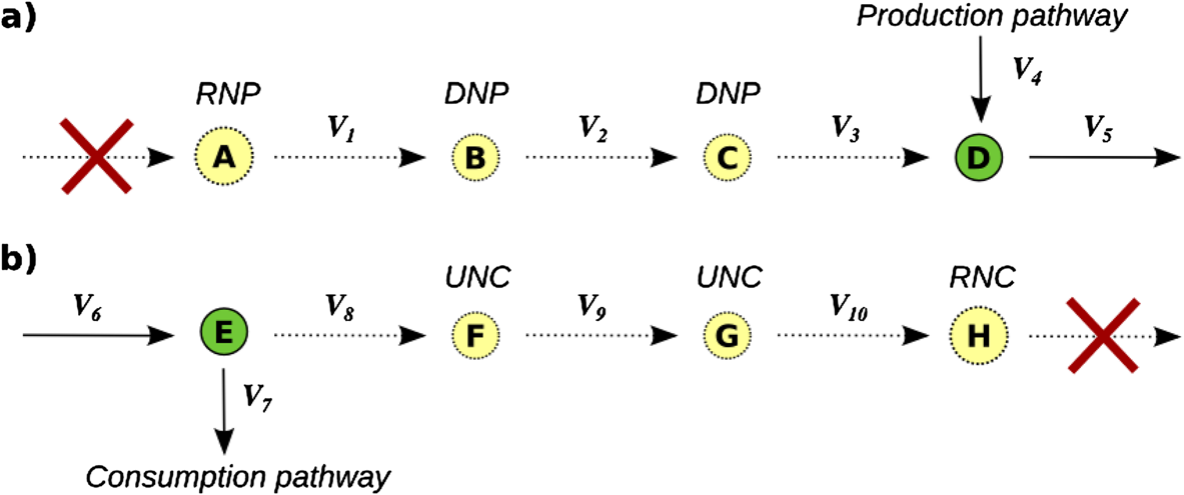
**Description of gap metabolites.** A schematic representation where the four classes of gap metabolites are shown as a consequence of missing reactions. Red crosses indicate the absence of some reaction. Dotted and continuous arrows represent blocked and non-blocked reactions, respectively. Yellow and green circles represent gap and non-gap metabolites, respectively. Metabolites are labeled according to its class. In **a)** the absence of a reaction, causes metabolite A to become a Root-Non-Produced metabolite (RNP) and this effect propagates downstream generating new gap metabolites (Downstream-Non-Produced, DNP) and blocked reactions. In **b)** the absence of reactions consuming H makes it a Root-Non-consumed metabolite (RNC) and this effect propagates upstream causing other metabolites to become Upstream-Non-Consumed (UNC), in a symmetric manner respect to case **a)**.

Detection of RNP and RNC can be conducted by simply scanning the rows of the stoichiometric matrix. However, the absence of flow through metabolites RNP (or RNC) could be propagated downstream (or upstream) by blocking reactions and thus, additional metabolites would become gaps (see Figure 1). Those metabolites that become a gap as a consequence of some RNP metabolite are termed Downstream-Non-Produced (DNP). In a symmetric way, Upstream-Non-Consumed (UNC) metabolites are defined as those metabolites that became a gap as a consequence of some present RNC metabolite [20]. In general, the detection of dead-end metabolites and blocked reactions is referred commonly as the gap finding problem [6,7,19,20].

Model’s gaps can be solved by adding one or more reactions that allow connecting a dead-end metabolite with other metabolites of the network, a process known as gap-filling [6,20]. In some cases, the incorporated reactions can be mapped into some coding gene. However, there are some situations where even if a set of candidate reactions that fill the gaps has been successfully predicted, it could not be possible to find the genes that code for these activities. In such cases the reactions are called orphan reactions. Methods to predict candidate genes to be assigned to orphan reactions have also been previously developed [21,22]. Thus, the reconstruction of metabolic models is an iterative process in which the CBM plays an important role to detect inconsistencies that should be resolved or curated in order to improve model formulation [23].

Automated methods for model curation have been previously developed (for a comprehensive review the reader is referred to [24]). In order to solve the gap-filling problem, an optimization-based method to identify the minimum number of reaction to be included in the model has been proposed by different authors [20,23]. These methods rely in the use of Mixed Integer Linear Programming (MILP) combined with universal reaction databases such as KEGG [25], BiGG [26] or MetaCyc [27]. Other proposed approaches are based on the use of experimental information to detect inconsistencies with model predictions that may suggest errors in model formulation [28,29].

Even though automated methods for metabolic network curation are of an undoubted help to improve model formulation, there may be situations where the manual inspections of a curator are still needed. This is certainly the case of the reconstruction of networks from genomes that suffer reductive evolution (*e.g.* intracellular bacterial symbionts) and code for minimized metabolisms. During the establishment of symbiosis, metabolic redundancies with the host can result in the loss of enzymatic steps in the endosymbiont network, leading to the emergence of obligate metabolic complementation. These shared metabolic abilities take the form of interrupted pathways when the endosymbiotic network is reconstructed. Thus, the problem of gap-filling is a complex decision making process where a visual representation of the inconsistencies can help to a model’s curator to understand how gap metabolites and blocked reactions are related and thus find the nature of these inconsistencies.

In this paper we present a method that combines the CBM with an algorithm to compute Connected Components over bipartite-graphs. The presented method allows the detection of the isolated sets of blocked and gap metabolites and the way in which these are interconnected in what we have termed unconnected modules (UM) (see *Identification of Unconnected Modules* in section Methods). Then, the analysis of each individual unconnected module simplifies and clarifies the visual representation, and hence can be used to decide how gaps should be filled during the curation of the model.

The availability of accurate GSM is especially relevant in the case of bacterial obligate (primary) endosymbionts since it is not possible the culture of these microorganisms and hence there is an intrinsic difficulty for obtaining direct experimental data from the system. In this case, modeling can serve as an appropriate proxy for functional characterizations. For instance, metabolic modeling has been successfully used in the case of *Buchnera aphidicola* (obligate endosymbiont of aphids) to evaluate the role of the endosymbiont and the host in nitrogen metabolism [30], in *Sodalis glossinidius* (facultative endosymbiont of tse-tse flies) to characterize intermediate steps during the reduction of the network as a result of the interaction with the host metabolism [31], and in *Blattabacterium cuenoti* (obligate endosymbiont of cockroaches) to better understand the striking conservation of the endosymbiont metabolism along the evolutionary time as well as its putative role in the nitrogen economy of the system [32]. In this paper we have used the GSM from *B. cuenoti (iCG238)* to test the proposed method of curation. Our study allows upgrading *iCG238* to a more accurate model version named *iMP240,* and it can be used as a guide for systems biology experimental explorations of the interaction between cockroaches and their endosymbionts.

## Methods

### Constraint-based modeling

The study of the structural properties of biochemical reactions network relies on the analysis of the stoichiometric matrix [33,34]. Let denote by *N* the stoichiometric matrix associated to a certain metabolic network with *m* rows and *n* columns corresponding to the number of metabolites and reactions, respectively. In the following *I* and *J* will refer to the set of metabolite indexes (rows) and reaction indexes (columns), respectively. Moreover, the set of reaction indexes *J* will be partitioned into two disjoint subsets: the set *J*_*INT*_ which contains the indexes of internal fluxes, *i.e.* the biochemical reactions that take place inside the cell, as well the transport reactions that operate between the cell and the surrounding medium. On the other hand, the set *J*_*EX*_ contains the indexes of the exchange fluxes, which are the auxiliary variables used to represent the rate at which certain metabolites are consumed or produced by the system [35]. There will be only one exchange flux per metabolite and these fluxes will be associated to the metabolites belonging to the extra-cellular compartment. By convention, the activity of the exchange fluxes is defined as positive or negative if the metabolite is produced or consumed by the system, respectively [35].

The CBM approach relays on the use of different kinds of constraints represented by mathematical equations to define the so called flux space *F*, *i.e.* the set of all flux distributions compatible with the given constraints [9,36,37]. The steady state condition is imposed over the mass balance equation of each metabolite of the network yielding the following homogeneous system of linear equations:

(1) N.v=0

where the vector *v* is a flux distribution compatible with the steady-state condition. Moreover, lower and upper bounds are imposed over each reaction to represent additional constraints. For instance, the thermodynamic constraints that make some reactions to be irreversible are represented by setting to zero the lower bound. Besides, the surrounding environment of a metabolic system can be modeled by setting bounds over the exchange fluxes. For example, if a given metabolite is available in the medium and thus can be consumed by the system, the lower bound of the corresponding exchange flux should have a negative value. The lower and upper bounds imposed over each flux can be written as the following systems of linear inequalities:

(2) $$v_{j}^{\text{lb}}\leq v_{j}\leq v_{j}^{\text{ub}}\;\forall \;j\varepsilon \;J$$

where *v*_*j*_ is the activity through the flux *j*, whereas $v_{j}^{\text{lb}}$ and $v_{j}^{\text{ub}}$ are its lower and upper bounds, respectively. Together, the homogeneous system of linear equations (1) and the system of linear inequalities (2) yields the mathematical representation of the flux space *F*, expressed as:

(3) $$F=\left\{v\;\varepsilon \;R^{n}:N.v=0,\;v_{j}^{\text{lb}}\leq v_{j}\leq v_{j}^{\text{ub}}\;\forall j\;\varepsilon \;J\right\}$$

### Blocked reactions

A reaction in a metabolic model is defined as blocked under a given medium condition if it cannot display a steady-state flux other than zero:

(4) $$j\;\varepsilon \;J_{\text{Blocked}}\Leftrightarrow v_{j}=0,\forall v\;\varepsilon \;F$$

where *J*_*Blocked*_ is the set of blocked reaction indexes. This set can be computed solving a set of linear programs, as proposed by Burgard *et* al. [38]. The approach consists in calculating the minimum and maximum flux value through each reaction of the system. When the maximum and minimum values found for a given reaction are both equal to zero, the reaction is said to be blocked under the defined medium condition. The formulation of the set of linear programs is the following:

(5) $$\begin{array}{l} \text{Min}/\text{Max}:v_{j}\;\forall \;j\varepsilon \;J\\ s.t.\\ N\cdot v=0\\ v_{j}^{\text{lb}}\leq v_{j}\leq v_{j}^{\text{ub}}\;\forall \;j\varepsilon \;J \end{array}$$

### Gap metabolites

Gap metabolites in GSM are defined as those vertexes of the network through which there can be no steady state flow [24]. While the detection of RNP and RNC metabolites (see the Introduction for a proper definition) is straightforward by scanning of each row of the stoichiometric matrix *N*, the case of detecting UNP and DNC metabolites cannot be accomplished by a simple inspection of the entries of *N*[20]. However, based on the definition of blocked reaction (4) we found a way to define gap metabolites that allow its identification in a straightforward manner.

*Definition: a metabolite ί ∈ I in a network under steady-state is a gap if and only if all the reactions in which its participate (either as reactant or as product) belongs to the set of blocked reactions J*_*Blocked*_*. This statement implies that there cannot exists a stationary flow through this metabolite.*

Thus, if we name the set of reactions in which a metabolite *ί* participate as:

(6) $$\sigma \left(i\right)=\left\{j\;\varepsilon \;J:N_{\text{ij}}\neq 0\;\right\}\;\forall i\;\varepsilon \;I$$

then, the set of gap metabolites *I*_*Gap*_ ⊆ *I* can be defined as follows:

(7) $$i\in I_{\text{Gap}}\Leftrightarrow \sigma \left(i\right)\subseteq J_{\text{Blocked}}$$

Hence, the detection of gap metabolites can be accomplished by finding the set *J*_*Blocked*_ and applying equations (6) and (7) for each metabolite. The given definition for a gap metabolite doesn’t make any distinction between the different classes of gap defined (*i.e.* RNP, RNC, UNP, DNC). Although there is no general procedure for the classification of gap metabolites as RNP, RNC, UNP, or DNC once they have been found, this is possible in simple cases. For example, if a gap metabolite *ί* is involved only in an irreversible reaction by which it is consumed, or if all the reactions in which this metabolite is involved are irreversible and in all it is consumed, then metabolite will be a RNP. A similar reasoning can we argued for the symmetric case, leading to the identification of a RNC. For more complex cases, a visual inspection of the underling *"Unconnected Module"* (see section: Identification of Unconnected Modules) may help to the classification.

### The coenzyme pseudo-gap problem

**Figure 2:**
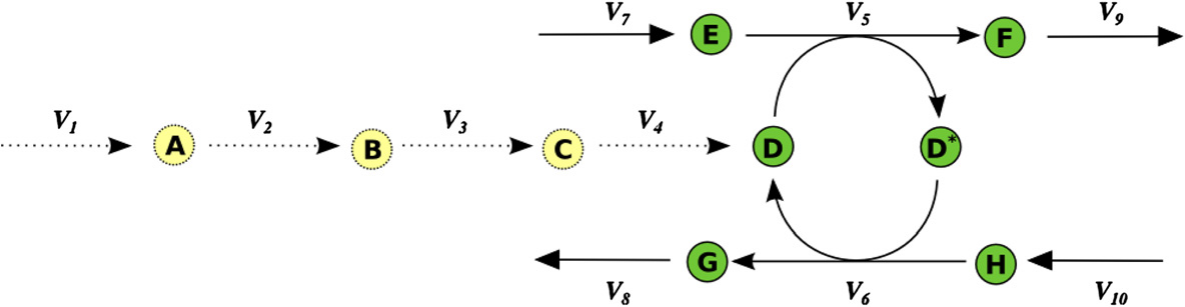
**Pseudo-gap metabolites.** Schematic representation of a situation where there is a metabolite not detected as gap (metabolite D) because of its participation in non-blocked reactions. However, the set of non-blocked reactions in which it participates forms a loop and there is no net production/consumption of the metabolite. As a consequence, the pathway of synthesis of D becomes blocked. The color and line codes are the same as in Figure 1.

In general gap metabolites can be identified using its relation to the set of blocked reactions *J*_*Blocked*_ as it was explained in previous subsection. However, there could be some special metabolites that are not "gap" under the definition given by (7). Nevertheless these metabolites may be the cause that certain reactions get blocked, as the example depicted in Figure 2 shows. The metabolite *D* is not a gap but the mass balances equation for D^*^ implies that *v*_5_ is equal to *v*_6_, and the mass balance equation for *D* implies *v*_4_ + *v*_6_ = *v*_5_. As a consequence of these relations *v*_4_ is restricted to zero, *i.e. v*_4_ is a blocked reaction. This effect propagates downstream to *v*_3_, *v*_2_ and *v*_1_. This is why we name *D* as a pseudo-gap metabolite. In order to unblock *v*_4_, which in term will unblock *v*_1_, *v*_2_ and *v*_3_, there must be added a sink for *D* or D^*^.

These kinds of situations may take place when the biosynthetic pathway of a coenzyme is present in a model, but there are no fluxes draining or degrading the coenzyme produced by this pathway. These metabolites may be involved in conserved moieties, and in such cases they will be consumed and regenerated in a cyclic manner. As a consequence they could not be detected as gap metabolites because they will participate in at least two active reactions. However, if the biosynthetic pathway for a coenzyme is included in a metabolic model, the net production of a coenzyme will not occur under steady state unless some flux consumes it. Hence, the reactions involved in the biosynthesis pathway may become blocked. A common approach to solve this problem is to include the coenzyme-like metabolite into the biomass equation or alternatively to introduce an exchange flux that can drain the metabolite out of the system. Either of these two situations is equivalent to the addition of the above-mentioned sink.

The way to detect the pseudo-gap metabolites may be summarized as follow. When an UM overlap with the biosynthetic pathway of a certain cofactor, two different situations can be found: the cofactor is included in the UM as a gap or it is not. If it is a gap, the cofactor must be included in the biomass equation in order to solve the UM (see "UM3 - Pyridoxal 5-phosphate biosynthetic pathway" under section Results and discussion as en example). If the cofactor is not included in the UM (*i.e.* is not a gap), then it is quite probable that the cofactor may be involved in a conservation relation which connect the cofactor to active reactions and for this reason is not detected as a gap under the definition given by equation (7). However, even not detected as gap, the cofactor could be the underlying cause that the UM become blocked, and for this reason was termed the cofactor a "pseudo-gap" as has been pointed out at the beginning of this section (as an example, see "UM1 - Menaquinol biosynthetic pathway", under section Results and discussion). The conservation relations can be detected by the analysis of the conserved moieties, which were calculated as previously described [33]. Finally, by adding the corresponding cofactor to the biomass equation the UM can be solved.

### Identification of unconnected modules (UM)

When analyzing the set of blocked reactions and gap metabolites of a metabolic model, it is common to find relations between both sets due to the fact that blocked reactions may be connected with other blocked reactions through gap metabolite. In some cases, blocked reactions are directly connected to a RNP/RNC metabolite. However, this may be not the case of other blocked reactions that may be not linked directly to a RNP or RNC metabolite. For example, as it can be seen in Figure 1a, reaction *v*_3_, although blocked, is not directly connected to a RNP metabolite (*A*), but indirectly through metabolite through a set DNC metabolites (*B* and *C*) and other blocked reactions (*v*_1_ and *v*_2_). Similarly occurs in the symmetric case (Figure 1b). As a consequence of these relations, it is possible to systematically establish how the blocked reactions are connected through the gap metabolites.

Any metabolic network can be represented as a directed bipartite graph by considering the sets of vertex *V = I*U*J*[39,40]. Then, a directed edge or arc will exist between a metabolite and a reaction if the metabolite participates in the reaction. The direction of the arc will be incident to the reaction if the metabolite is a reactant and incident to the metabolite if it is a product. In the following, the graph associated to a metabolic network will be referred as the metabolic graph.

Once the metabolic graph is constructed it is possible to consider any possible sub-graph by selecting a pair of subsets *I*^'^ ⊆ *I* and *J*^'^ ⊆ *J* of metabolites and reactions respectively. In particular, it is possible to consider the sub-graph defined by the subset of vertex *V = I*_*Gap*_U*J*_*Blocked*_. This sub-graph will contain the relation that exists between gap metabolites and blocked reactions. Moreover, the set of connected components can be computed over this graph. In this context, each connected component can be interpreted as a "module" of the metabolism that becomes inactive or unconnected, possibly as a consequence of model inconsistencies, such as the presences of a set of RNP/RNC metabolites. For this reason the set of connected component will be referred as an *Unconnected Modules* (UM).

In simple cases, the reason that causes a certain UM to be unconnected from the rest of the network may be found by visual inspection of the graph representing the UM. In such cases connectivity restoring of the RNC and RNP metabolites present in the UM may, in general, solve the problem of the UNC and DNP metabolites. The set of elementary operations that can be applied to solve UMs has been discussed by Kumar and collaborators [20], and it includes: addition of biochemical reactions of transport, incorporation of exchange fluxes and relaxation of irreversibility constraint of some reactions. More complex situations may include cases such as the pseudo-gap problem described in the previous section.

### Flux balance analysis

The Flux Balance Analysis (FBA) is an approach that combines the description of the flux space *F* defined by equation (3) with optimization techniques to find a flux distribution *v* that maximizes the growth rate [17,37,41]. This problem can formulated as a linear program and can be solved with standard techniques of Linear Programming (LP).

(8) $$\begin{array}{l} \text{Max}:v_{\text{Biomass}}\\ s.t.\\ N\cdot v=0\\ v_{j}^{\text{lb}}\leq v_{j}\leq v_{j}^{\text{ub}}\;\forall \;j\varepsilon \;J \end{array}$$

where the flux *v*_*Biomass*_ represents the growth rate of the organism. This flux, also refereed as the biomass equation, includes all the metabolites that are biomass components, in its specific proportions [42,43].

### In-silico knockout experiments

The fragility analysis of a network was performed by simulating knockout experiments for each metabolic gene included in a GSM. An *in-silico* knockout experiment for a given gene consists in bound to zero the flux for each reaction coded by the gene, which are inferred through the GRP association table. After so, FBA is used to find the maximal value of the biomass reaction under the genetic perturbation. If the optimal value of biomass reaction is lower than a certain threshold, the knockout is said to be lethal (*i.e*. essential), otherwise the gene is not essential. The procedure is performed over all the genes in the model.

### Minimal medium prediction

The minimal medium may be defined as the smaller set of metabolites that should be present in the medium condition in order a feasible flux distribution *v* to exist, with a biomass production rate greater than zero. The minimal medium was calculated by solving a MILP algorithm as previously described [44-46]. The MILP algorithm is the following:

(9) $$\begin{array}{l} \text{Min}:\underset{j\;\varepsilon \;J_{\text{EX}}}{\sum }y_{j}\\ s.t.\\ N\cdot v=0\\ v_{j}^{\text{lb}}\leq v_{j}\leq v_{j}^{\text{ub}}\;\forall \;j\varepsilon \;J_{\text{INT}}\\ v_{\text{Biomass}}\geq v_{\text{Biomass}}^{\text{lb}}\\ v_{j}^{\text{lb}}y_{j}\leq v_{j}\;\forall \;j\varepsilon \;J_{\text{EX}}\\ y_{j}\;\varepsilon \;\left\{0,1\right\}\;\forall \;j\varepsilon \;J_{\text{EX}} \end{array}$$

The algorithm requires the incorporation of a set of binary *y*_*j*_ variables, one for each exchange flux *j* ε *J*_*EX*_. Moreover, a set of constraints that relates each binary variable with its corresponding exchanges flux should be incorporated to the problem. Then, whenever a binary variable takes the zero value, the corresponding exchange flux is constrained to take a value greater or equal than zero. Additionally, the lower bound corresponding to the biomass production flux should be set to a cutoff value greater than zero in order to guarantee a positive growth rate. Finally, the optimization target is defined in such a way that minimizes the number of active exchange fluxes with a negative value. Due to the fact that each exchange flux is related with one extracellular metabolite this is equivalent to find the minimal set of metabolites that the system must consume in order to produce a biomass flux greater than zero.

### Detection of reaction subsets

A *Reaction Subsets* (RS) [47,48] or *Full Coupling Sets*[38] in a stoichiometric network is a group of reactions that operate together in fixed flux proportions for any flux distribution. Due to the fact that the relations between enzymes and reactions are not always biunivocal, the reaction subset does not always match the concept of *Enzyme Subset* previously introduce by Pfeiffer *et* al. [49], and for this reason the term Reaction Subset seams more appropriate [48]. The RSs are structural invariant of network and for that they are independent on the kinetic parameters of the system. Moreover, they shed valuable information that may help to understand how the network is regulated [50].For these reasons the concept RS is important for the analysis of metabolic networks.

In order to compute reaction subset the following pre-processing steps were applied to the network. First, rows and columns corresponding to gap metabolites and blocked reaction respectively were removed from the stoichiometric matrix. As it was previously described [38], the constant biomass composition imposed by the stoichiometry of the biomass equation was relaxed by removing the corresponding column, while allowing each biomass component to be drained from the system in an independent way. Then, the identification of RS was done by using the algorithm described in [49].

### Computational tools

Constraint-Based analysis was performed using the python-based toolbox COBRApy [51]. LP and MILP problems were solved using the Gurobi Solver [52] acceded through COBRApy. Identification of conservation relations was done by computation the set of extreme rays using the Polco package [53]. Identification of RS was done by using an implementation based on Python [54,55] of the algorithm described in [49]. The detection of the connected components of a graph was done using the implementation of the algorithm available in the iGraph library [56]. Graphs were drawn using the yEd Graph Editor [57]. All computation was done on a desktop computer with an Intel® Core™ i7 CPU 950 processor, with 23.5 GiB, running under Fedora 17 Linux OS.

## Results and discussion

The first step in the analysis of the GSM of *B. cuenoti iCG238* was to find the sets *J*_*Blocked*_ and *I*_*Gap*_ of blocked reactions and gap metabolites, respectively. The results showed that 69 reactions over a total of 419 (~16%) are blocked under any medium condition. Using this information a set of 58 metabolites over a total of 364 (~15%) were detected as gaps. A bipartite graph representation of the metabolic network was constructed, and the sub-graph defined by the subset of vertex *I*_*Gap*_ U *J*_*Blocked*_ was selected (see Identification of Unconnected Modules in section Methods). Computation of connected components over this sub-graph allows identifying 10 UM. After so, each gap metabolite was classified in one of the four categories: RNP, RNC, UNC and DNP. A description of each different UM found in *iCG238* is summarized in Table 1, which was sorted according to the number of reactions included in each UM.

**Table 1:** Description of UMs

**UM**	**Related to subsystem**	**No. reactions**	**No. metabolites**	**RNP**	**RNC**
1	Menaquinol Biosynthesis	23	21	Mev, 2ombzl	2ommbl
2	Nucleotide Salvage Pathway	22	15	-	Hxan, xan, r1p, 2dr1p, thym, ura
3	Pyridoxal 5-phosphate Biosynthesis	7	6	-	4hthr
4	Lipopolysaccharide Biosynthesis	4	4	-	u3hga
5	Siroheme Biosynthesis	4	4	-	uppg3
6	Arginine and Proline Metabolism	2	2	-	1pyr5c
7	Transport, Extracellular (Fe^2+^)	2	2	-	Fe^2+^
8	Transport, Extracellular (K^+^)	2	2	-	K^+^
9	Superoxide Dismutase	1	1	O_2_^–^	-
10	Acyl-Carrier Protein Synthase	1	1	apoACP	-
	Total	68	58	8	4

UM identified in *B. cuenoti iCG238* GSM. Metabolite abbreviations: Mev (mevalonate), 2ombzl (2-Octaprenyl-6-methoxy-1,4-benzoquinol), 2ommbl (2-Octaprenyl-3-methyl-6-methoxy-1,4-benzoquinol), hxan (Hypoxanthine), xan (Xanthine), r1p (Ribose-1-phosphate), 2dr1p (2-deoxy-D-Ribose-1-phosphate, thym (Thymine), ura (Uracil) 1pyr5c (1-Pyrroline-5-carboxylate), 4hthr (4-Hydroxy-L-threonine), u3hga (UDP-3-O-(3-hydroxytetradecanoyl)-D-glucosamine), uppg3 (Uroporphyrinogen), apoACP (apoprotein [acyl carrier protein]). The relation between an UM and a subsystem was established according to the most frequent subsystem associated to the reactions of the UM.

When analyzing the reactions participation of each UM, it was found that in many of the cases the set of reactions belonging to an UM overlapped with known biochemical subsystems (or metabolic pathways). For example, all the reaction included in UM1 belongs to the menaquinol biosynthetic pathway. It is worth to note that in some cases UMs could be composed by an isolated reaction as the cases of UM9 and UM10. After the identification of all UM applying the proposed approach, each sub-graph was drawn independently. Visual inspection of each graph was done to detect possible inconsistencies in the network formulation that may help to carry out the manual curation of a GSM.

### UM1 - *Menaquinol biosynthetic pathway*

**Figure 3:**
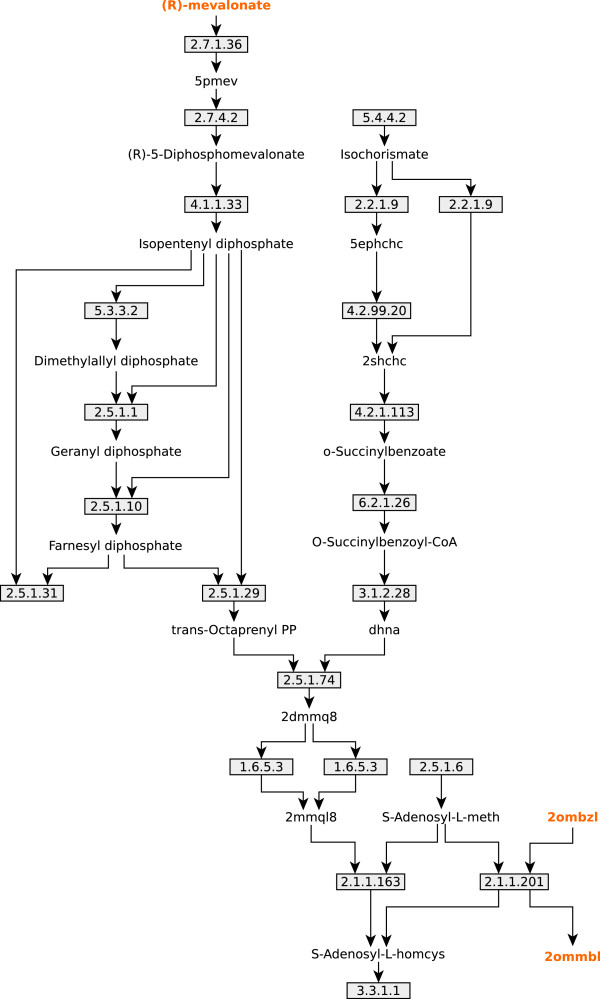
**UM1 scheme.** Schematic representation of the biggest UM found in *B. cuenoti iCG238* model, which includes all the reactions belonging to the Menaquinol Biosynthetic Pathway. Metabolites are represented by name labels and colored accordingly to its category (yellow for RNP and RNC; black for DNP and UNC); reactions are represented as squares with its associated EC number.

The biggest UM found in the metabolic model (UM1) was first analyzed. Figure 3 shows the graph that represents the UM. A visual inspection of these Figure shows that UM1 includes all the reactions and metabolite that conforms the menaquinol biosynthetic pathway. The Figure also shows that from the 21 gap metabolites included in the UM, two are RNP metabolites (mevalonate*,* 2-Octaprenyl-6-methoxy-1,4-benzoquinol), and one is an RNC metabolite (2-Octaprenyl-3-methyl-6-methoxy-1,4-benzoquinol), while the remaining metabolites are then upstream/downstream gap metabolites.

Mevalonate is a precursor for the biosynthesis of isopentenyl diphosphate that in turn is involved in the biosynthetic pathway of many cofactors such as menaquinol and 2-demetyl-menaquinol. An inspection of the current genome annotation of *B. cuenoti*[58] was done to look for candidate genes coding for enzymes belonging to the mevalonate biosynthetic pathway. However, none of those genes were identified indicating a plausible partial loss of the mevalonate biosynthetic pathway. As a consequence of this putative lost trait, mevalonate should be hypothetically imported from the environment (*i.e.* the insect host), a situation that would suggest a new case of metabolic complementation between the bacterial endosymbiont and its host. In favor of our hypothesis we point out that the mevalonate pathway plays a key role in insect metabolism as the precursor of juvenile hormone (JH) and it is active in the fat body of *B. germanica* ( [59] and references therein). Indirect evidence of the ability of mevalonate to diffuse and reach the endosymbiont is given by feeding experiments using mevalonate as a precursor of JH synthesis in the corpora allata ( [59] and references therein).

Moreover, the locus tag BLBBGE_110 has been annotated as a homolog to *ubiE*[58], which codes for an enzyme, a C-methyltransferase, that catalyzes reactions in both ubiquinone (Q) and menaquinone (MK) biosynthesis [60]. In Q biosynthesis, UbiE catalyzes the conversion of 2-octaprenyl-6-methoxy-1,4-benzoquinone to 2-octaprenyl-3-methyl-6-methoxy-1,4-benzoquinone (EC 2.1.1.201). In MK biosynthesis, UbiE catalyzes the conversion of demethylmenaquinone to menaquinone (EC 2.1.1.163). While the genome of *Blattabacterium* has putative genes that code for the remaining activities for the MK biosynthesis, it has not any other annotated gene that accounts for the activities of the Q biosynthesis. As a consequence the activity EC 2.1.1.201 has no biological meaning in the metabolic network of *B. cuenoti* and for that reason it should not be included in the model.

Assuming a case of metabolic complementation between the bacteria and its host where mevalonate is a metabolite supplied by the host, a transport flux that allow the uptake of mevalonate by the cell was incorporated to the model. After so UM where recomputed just to find out that UM1 was still non-functional. A new inspection of the graph showed that there were two "dead end" reactions (EC 2.5.1.31 and EC 3.3.1.1) for which no products were included as RNC metabolites. A closer inspection of these reactions showed the following: reaction EC 2.5.1.31 produces undecaprenyl diphosphate, a metabolite that works as a coenzyme in the biosynthesis of murein, whereas reaction EC 3.3.1.1 is the last step in the biosynthesis of menaquinol, which is a known coenzyme that operates as an electron carrier. As it is explained in section Methods (The coenzyme pseudo-gap problem), both metabolites were then included in the biomass equation. After so, UM1 was completely solved, meaning that all reactions got unblocked (Additional file 1: Figure S1).

### UM2 - *Nucleotide salvage pathway*

**Figure 4:**
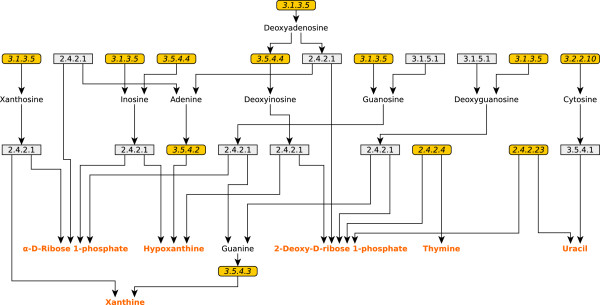
**UM2 scheme.** Schematic representation of the UM2 that corresponds to the Nucleotide Salvage Pathway. Reactions and metabolites are represented as in Figure 1. However, in this case the reactions with no gene association (*i.e.* orphan reactions), or wrong EC assignations, are represented with rounded rectangles and highlighted in yellow.

In this case, the set of 22 reactions contained in the UM belongs to the nucleotide salvage pathway. The set of genes coding for these activities was analyzed and it was found that 12 of the reactions are orphans, *i.e.* they do not have an associated coding gene (see Figure 4). The remaining 10 reactions were grouped according to its coding gene and this showed that 3 genes code these activities in the following way: seven reactions, that correspond to the activity 2.4.2.1, are assigned to gene BLBBGE_377 [GenBank: CP001487], annotated as purine-nucleoside phosphorylase; two reactions defined by the activities EC 3.1.5.1 are associated to gene BLBBGE_612 [GenBank: CP001487]; finally, the activity EC 3.5.4.1 is assigned to the gene BLBBGE_353 [GenBank: CP001487]. Due to the great number of orphan reactions contained in UM2 and based on the fact that all of these reactions are predicted as blocked, the first step to analyze this UM was to remove its orphan reactions. After so, the set of UMs were recalculated to evaluate the impact of these changes. It was found that the UM splits into two UMs: one of them formed by all the reactions associated with the activities EC 2.4.2.1 together with the two reactions associated with activity EC 3.1.5.1; the other one was composed by the isolated reaction EC 3.5.4.1. In both situations, most of the metabolites involved in the UMs were classified as RNP or RNC (Additional file 1: Figure S2b).

With the purpose of evaluating the functional assignment of these three genes, a close inspection of the genome annotation was done. First, it was found that the annotation of BLBBGE_612 [GenBank: CP001487] does not have strong evidence supporting that this gene code for the activity EC 3.1.5.1. Thus, considering the lack of information supporting the association between the gene and the activity and taking into account that the model predicts that both reactions associated with activity EC 3.1.5.1 are blocked, it is more plausible to assume that the activity does not take place in the metabolism of the endosymbiont. Second, the association of the enzyme activities EC 2.4.2.1 and EC 3.1.5.1 to genes BLBBGE_377 and BLBBGE_353 [GenBank: CP001487], respectively, is supported by the genome annotation. However, the activity EC 2.4.2.1 appears to have a broad substrate specificity and seven different reactions associated with this activity are included in model *iCG238,* all of them predicted to be blocked because their direct connection to an RNP, an RNC or to both. In the case of the activity EC 3.1.5.1 the reaction becomes isolated being one of its substrates and one of its products a RNP and a RNC metabolites, respectively. Due to the complexity of this situation and in the absence of experimental evidence that could help to solve these metabolic puzzles it was not possible to find the role of these activities in the metabolism of the bacterium, and thus the set of genes with its associated activities were excluded from the model until new experimental data shed some light over this problem.

### UM3 - *Pyridoxal 5-phosphate biosynthetic pathway*

The seven reactions involved in this sub-graph correspond to the complete Pyridoxal-5-phosphate pathway (Additional file 1: Figure S3). Pyridoxal-5-phosphate, also called Vitamin B6, functions as a cofactor of different enzymes involved, among others, in transamination reactions required for the synthesis and catabolism of amino acids. Due to its importance in metabolism, this metabolite was included in the biomass equation in the same way that it has been done with other cofactors and coenzymes. As a result all the reactions in the UM become unblocked. It is worth to note that animals, in particular insects, do not possess any biosynthetic pathway for pyridoxal-5-phosphate, and for this reason they need to take it from their diet in order to survive [61]. As a consequence *B. cuenoti* may provide its host with this metabolite, suggesting another case of possible metabolic complementation.

### UM4 - *Lipopolysaccharide biosynthetic pathway*

The UM4 is composed by a linear chain of four reactions that are related to the biosynthetic pathways of different membrane lipids. The first two reactions are involved in the palmitate biosynthetic pathway. However, these reactions are not biochemically defined, but they are in turn the condensation of a set of reactions. For example, the first reaction labeled as C120SN is the net sum of 19 activities that produce dodecanoyl-ACP from acetoacetyl-ACP. Moreover, the reaction KAS16 is also the condensation of the activities EC 2.3.1.41 and EC 1.1.1.100 (Additional file 1: Figure S4). The other two reactions of the UM are the first and second steps of the Lipid IV_A_ biosynthetic pathway. UM4 was reformulated by decomposing the reactions C120SN and KAS16 in its corresponding activities. After so, the structure of the UM was analyzed to find the following. First, the activities EC 4.2.1.58 and EC 4.2.1.59, seem to be orphan in *ICG238.* Second, the activities assigned to the Lipid IV_A_ biosynthetic pathway were all orphan except for the activity EC 3.5.1.108, which was assigned to the gene BLBBGE_037 [GenBank: CP001487]. This scenario suggests that this pathway may be absent, and thus it could be the consequences of an error in the annotation of BLBBGE_037 [GenBank: CP001487]. Indeed, it was found that the set of ortholog genes identified in the genome of other sequenced genomes from diverse *B. cuenoti* strains have been annotated as coding for the activities EC 4.2.1.58 and EC 4.2.1.59. As a consequence, the annotated activity of BLBBGE_037 [GenBank: CP001487] was changed from EC 3.5.1.108 to EC 4.2.1.58 and EC 4.2.1.59. The remaining orphan activity present in the Lipid IV_A_ pathway was also removed from the model because it is assumed that this pathway is not present in the metabolism of *B. cuenoti.*

### UM5 - *Siroheme biosynthetic pathway*

A linear chain of four reactions composes the UM5, where the last reaction produces uroporphyrinogen III, which was found to be a RNC (see Table 1). This metabolite acts as substrate in the biosynthesis of siroheme, a prosthetic group which catalyzes the reduction of sulfite to sulfide and of nitrite to ammonia in the assimilation and dissimilation of sulfur and nitrogen compounds [62].

After an inspection of *B. cuenoti* genome annotation, it was found that all the coding genes of the biosynthetic pathway of siroheme are present. However, they had not been previously included in the metabolic model. The pathway consists in four reactions (arranged in a linear pathway) coded by two genes: BLBBGE_281 [GenBank: CP001487] that codes the enzyme with activity EC 1.3.1.76 and the gene BLBBGE_278 [GenBank: CP001487] which codes an enzyme that catalyzes activities EC 2.1.1.107 and EC 4.99.1.4.

Since siroheme is an important cofactor involved in sulfur and nitrogen metabolism, the cell would have to be able to maintain certain pool of this cofactor. Hence, during the bacterial growth phase, the organism will need some production of siroheme. In order to take into account this fact, siroheme was included into the biomass equation. After including this modification to the model, FBA was applied to find a metabolic state that maximizes biomass production. As expected, it was found that the four reactions included in UM5 and the four new reactions added to the model showed non-zero flux under optimal state (Additional file 1: Figure S5).

### UM6 - *Proline biosynthetic pathway*

In this case two reactions were found: N-acetylornithine deacetylase coded by gene BLBBG_320 (with no corresponding EC number) and L-glutamate 5-semialdehyde dehydratase annotated to be spontaneous. Both reactions were annotated as belonging to the proline biosynthetic pathway. In this scenario suggest that the pathway only lacks the last reaction step in order to be able to produce proline (Additional file 1: Figure S7). However a closer look to the annotation of gene BLBBG_320 shows that it has been assigned to code the following 3 activities:

1. *N-Acetyl-L-glutamate 5-semialdehyde + H*_*2*_*O → Acetate + L-Glutamate 5-semialdehyde*

2. *N-acetyl-L-ornithine + H*_*2*_*O → L-ornithine + acetate*

3. *N-succinyl-L,L-2,6-diaminopimelate + H*_*2*_*O → L,L-diaminopimelate + succinate*

Reaction (1) could not be found in the BRENDA database. However it was found as an entry in BiGG database [26]. For these reason it is not clear whereas the reaction has been biochemically characterized or not. In addition, experimental results show that proline is the most abundant amino acid in the cockroach’s hemolymph [63] supporting the hypothesis that proline is provided by the host. As a consequence, considering UM6 as an annotation error seems a more plausible hypothesis and thus these activities were not included in the new version of the metabolic model.

### UM7–UM10 - The case of isolated reactions

In the cases of UM7 and UM8, both of them correspond to transport reactions associated to two different ions: Fe^2+^ and Mg^2+^ and, the associated exchange fluxes. Both ions are included in the biomass equation as described by other authors [19,42] and in this way both UMs got unblocked (Additional file 1: Figure S6). The isolated reaction that defines UM9 is superoxide dismutase (EC 1.15.1.1), a reaction that together with the activity EC 1.11.1.6 works as a detoxification pathway against free radicals such as superoxide and hydrogen peroxide (Additional file 1: Figure S7b). Both activities have an associated coding gene and there is also experimental information suggesting the aerobic character of *B. cuenoti*[32]. Taking together these facts it is expected that superoxide dismutase plays an important role in the metabolism of the endosymbiont. The graph analysis showed the superoxide as a RNP metabolite. The explanation of the previous finding rely in the fact that the processes of free radical formation, *e.g.* as by product of aerobic respiration, is out of the scope of the model and hence the model does not include any reaction producing superoxide.

UM10 is also a case of an isolated reaction (EC 2.7.8.7) which catalyzes the activation of the apoprotein into acyl-carrier-protein (ACP), been this product a highly conserved carrier of acyl intermediates, important for fatty acid synthesis (Additional file 1: Figure S7c). The process of protein biosynthesis is not included in the metabolic model of *B. cuenoti* (neither the DNA nor RNA biosynthesis) and for that reason the model doesn’t include any reaction producing the apoprotein. As a consequence, the apoprotein becomes a RNP metabolite that blocks the activity EC 2.7.8.7. As in the case of UM9, some metabolite (*e.g*. the apoprotein) is produced by a metabolic process that is out of the scope of the model and thus appears as a dead-end.

### Model update

The curation process described in the previous section resulted in the removal of 6 genes associated to 6 reactions from *iCG238* and the addition of 8 new genes corresponding to a total of 9 reactions. Thus, the new model version has two more genes (240) and for this reason has been named as *iMP240*. Moreover, the reassignment and the inclusion of activities as well as the removal of orphan activities lead to elimination of 73 reactions (71 reactions plus 2 exchange fluxes) from *iCG238* and the inclusion of 59 new reactions (56 reactions plus 3 exchange fluxes) in *iMP240* (see Additional file 2). Since no experimental data was available for *Blattabacterium* Bge, chemical composition of *E. coli,* adapted from [19], was used. In particular, the stoichiometric coefficients of the new cofactor included to the biomass of *iMP240*, were those found in *E. coli* model *iJO1366*. Even if *Blattabacterium* and *E. coli* are phylogenetically very distant organisms, it is worth to note that these coefficients are approximations of the order of magnitude meant to capture the needs of an organism during growth in a qualitatively fashion. For a detailed comparison between both models see Additional file 3.

### Comparative analysis of the reaction subsets

The curation process involved addition and removal of reaction and metabolites as well as changes in the formulation of the biomass equation. These changes affected the stoichiometric matrix, and then resulted in different structural properties of the network. In particular, we have analyzed the organization of the Reaction Subsets (RS).

Table 2 summarizes the number of RS identified for the two models, as well the number of reaction within each RS. The Jaccard index was used as a measure of similarity between the RS from the two models. This index was calculated for each pair of RS as the cardinality of the intersection over the cardinality of the union between both RSs. Thus its value is bounded between 0 and 1. The higher the index value, the higher is the number of reaction shared by both RS.

**Table 2:** Comparison of RS

**No. reactions in RS**	***iMP240***	***iCG238***	**J**_**1.0**_	**J**_**.75**_
2	27	28	21	--
3	8	6	3	1 (+1)
4	2	4	1	--
5	4	3	2	--
6	3	3	2	--
7	3	1	1	1 (+1)
8	--	1	--	--
9	4	3	3	--
10	1	1	--	1 (+1)
11	--	1	--	--
13	1	--	--	--
17	--	1	--	--
18	1	--	--	1 (-1)
20	1	--	--	--

The first column indicates the number of reactions that belong to a RS. The second and third columns indicate how many RS with a given number of reaction are in model *iMP240* and *iCG238*, respectively. The fourth column shows how many RS pairs are equal in both models and for a given number of reactions. In the last columns, the number out of the parenthesis indicate how many RS with a Jaccard index greater than 0.75 have been found, taking as the reference point the model *iMP240*. The number in parenthesis indicates the difference of reactions between a pair of RS in the following way: a positive number means that the RS in *iCG238* has this number of additional reactions whereas a negative indicate the opposite.

The major difference found in the reorganization was the presence of a RS composed by 19 reactions present in *iMP240* but not found in *iCG238*. This difference is due to the fact that model *iCG238* has all these 19 reactions condensed in a single step (Additional file 1: Figure S4). This is also the case of both the RS that contains 13 reactions and one of the RS composed by 7 reactions founded in *iMP240*. Moreover, there is another RS of 7 reactions only present in *iMP240* that corresponds to the Pyridoxal Biosynthetic Pathway. This pathway was blocked in *iCG238,* and hence cannot be detected as an RS. Additionally, there are three RS almost equal in both models, but differing in only one reaction. For example, the RS of 18 reactions in *iMP240* corresponds to the RS present in *iCG238* that contains 17 reactions. Despite the differences described above, no major changes were found in the organizations of RS between both models.

### Comparison of minimal medium

The *in-silico* minimal set of compounds needed for the endosymbiont in order to produce all biomass components was predicted for both model versions (*iCG238* and *iMP240*), using an optimization algorithm (see *Minimal Medium Prediction* in Methods section). Table 3 shows the results. As it can be seen, all the metabolites included in the minimal medium predicted for model *iCG238* are included in the minimal medium predicted for *iMP240*, except for (S)-Dihydroorotate. This metabolite is a precursor in the biosynthesis of pyrimidines. The reason for this difference is that *iCG238* did not include the activity EC 3.5.2.3, which catalyzes the conversion of N-carbamoyl-L-aspartate into (S)-Dihydroorotate, and thus the model predicts that (S)-Dihydroorotate should be uptaken from an external source. The activity EC 3.5.2.3 has been found to be coded by the gene BLBBGE_317 [GenBank: CP001487], and its inclusion in the new model predicts that (S)-Dihydroorotate can be produced by the metabolism of the endosymbiont.

**Table 3:** Minimal medium

**Medium components**	***iMP240***	***iCG238***
Thiamin	Required	Required
Nicotinate	Required	Required
Sodium	Required	Required
L-Glutamine	Required	Required
Sulfate	Required	Required
(R)-Pantothenate	Required	Required
Phosphate	Required	Required
L-Asparagine	Required	Required
L-Proline	Required	Required
Glycine	Required	Required
O_2_	Required	Required
(S)-Dihydroorotate	--	Required
(R)-Mevalonate	Required	--
Glycerol	Required	--
Porphobilinogen	Required	--
Fe^2+^	Required	--
K^+^	Required	--
Mg^2+^	Required	--

Comparative table showing the *in-silico* predicted minimal medium for the new version model *iMP240* and previous version *iCG238*.

The predictions using the model *iMP240* also suggest six new compounds that need to be present in the medium in order for the organism to be able to grow. These sets of metabolites include: i) mevalonate, which is needed to synthesize menaquinol and 2-demetyl-menaquinol; ii) glycerol, which is phosphorylated by the activity EC 2.7.1.30, and used in the biosynthesis of phosphatidylglycerol species. In model *iGC238* glycerol-3-phosphate is produced from dihydroxyacetonephosphate through the reaction EC 1.1.5.3 operating in reverse sense. However, it was not possible to found any evidence suggesting that the reaction EC 1.1.5.3 could operate in a reversible manner. Hence, if the reaction is considered as irreversible, then the cell cannot produce glycerol-3-phosphate. Then glycerol should be uptaken from the medium and phosphorylated inside the cell. iii) Porphobilinogen that is converted into hydroxymethylbilane, the first precursor in the synthesis of siroheme, which in turn requires Fe^2+^; iv) the case of K^+^ and Mg^2+^ are trivial: these ions become required in the minimal medium after their inclusion into the biomass equation.

### Network fragility

**Figure 5:**
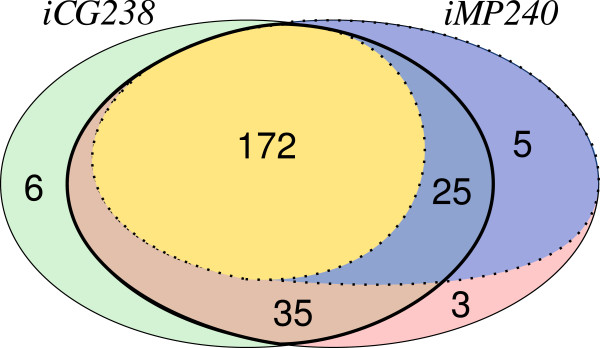
**Differences between models *iCG238 *and *iMP240.*** Venn diagram representing the main differences between models *iCG238* and *iMP240*. The sets drawn with a thin continuous line represents the genes included in each model. The set delimited by thick solid line represents the set of genes present in both models, *i.e.* the intersection. Finally, sets defined by dotted lines indicate genes predicted as essential by the *in-silico* simulations over each model.

The set of essential metabolic genes were computed by an *in-silico* analysis of the model *iMP240*. These results were then compared with the set of essential genes previously predicted for model *iCG238*[32]. Figure 5 summarizes the results of the comparative analysis between both models. The set of 172 genes (~72.3%) predicted as essential in *iCG238* remains as essential in *iMP240*. In addition, a set of 30 genes was predicted as essential in *iMP240*, resulting in total of 202 metabolic genes predicted as essential (~84%). As it is depicted in Figure 5, 25 of those 30 genes were present in the previous version of the model (*iCG238)*, while the remaining 5 are new genes.

This increase in the predicted network fragility obtained after model curation is explained, in greater extent, due to the addition of new components to the biomass equation. For example, the genes coding for the biosynthetic pathway of menaquinol where predicted as non-essential in model *iCG238* because the cofactor was not included in the biomass equation. However, the cofactor must be essential to the organism, due to its strict aerobiosis, and then it should be included as a biomass component. As a consequence most of the genes coding the biosynthetic pathway of menaquinol are predicted as essential in the new scenario. Moreover, the 5 new genes included in *iMP240* that are predicted as essentials, include two genes that code for three steps in the biosynthesis of siroheme, two genes that code for the transport of nicotinamide and Mg^+2^, and the gene that encode Ftsl, an essential cell division protein.

## Conclusion

In this paper we have introduced a general definition of gap metabolite that allows its detection in a straightforward manner, even for the cases of upstream-non-produced and downstream-non-consumed metabolites. Moreover, a method for the detection of *Unconnected Modules* (UM), defined as isolated sets of blocked reactions connected through gap metabolites have been proposed. The visual representation of UM can shed useful information that may help the model’s curator to solve inconsistencies. Furthermore, the present approach can be combined with existing tools in order to find a set of model modifications that solves the inconsistencies and thus improves model’s predictions.

This method was applied to the curation of the GSM of the cockroach endosymbiont *B. cuenoti iCG238.* In this way every blocked reaction detected in the model was successfully unblocked or alternatively removed, in those cases where there was not enough information supporting the existence of such reactions. As an example, those reactions found as blocked and with no gene association were excluded. Moreover, new reactions were added to the model based on the careful revision of the genome annotation that allows the identification of gene functions previously not included, as well as the incorporation of new compounds into the biomass equation. As a consequence of model curation a new GSM version of *B. cuenoti,* named as *iMP240,* is proposed. The impact of these modifications, with respect to some structural properties of the networks, was analyzed by performing different *in-silico* analysis over each model’s version.

As a final commentary, the method here presented can be considered as a semi-automatic approach that has the advantage of allowing a quick representation of the gaps of the model but that needs the supervision of an expert in the biology of the studied organism. This issue may be seen as a drawback of the method but, as in the case of automatic genome annotation, there is a trade-off between the degree of automation of the metabolic reconstruction and the quality of the generated model.

## Abbreviations

RNP: Root-non-produced; RNC: Root-non-consumed; DNP: Downstream-non-produced; UNC: Upstream-non-consume; UM: Unconnected module; FBA: Flux balance analysis; CBM: Constraint based modeling; GSM: Genome scale model; RS: Reactions subset; LP: Linear programming; MILP: Mixed integer linear programming; GPR: Gene-protein-reaction; EC: Enzyme commission number; TC: Transport commission number.

## Competing interests

The authors declare that they have no competing interests.

## Authors’ contributions

JP, FM and MPL presented the original idea of the work and designed the study. MPL conceived the algorithmic procedure, implemented the code, and carried out the *in-silico* experiments. All authors contributed in designing research, analyzing the data, and writing the paper. All authors have read and approved the manuscript.

## Supplementary Material

Additional file 1 **Schematic representation of UMs.** The file contains schemes and a brief description of each UM. Click here for file

Additional file 2 **New GSM model of B. cuenoti iMP240.** Spreadsheet with the model description. The file contains three sheets corresponding to Metabolites, Reactions, and Exchange Fluxes, respectively. Click here for file

Additional file 3 **Comparative table iCG238 vs iMP240.** Spreadsheet describing the main differences between both model versions. The file contains three sheets: the first one includes the genes added as well the genes that were removed; the second one presents the removed reactions; and the third one shows the set of added reactions. Click here for file
