# Supplementary material for: Solving gap metabolites and blocked reactions in genome-scale models: application to the metabolic network of Blattabacterium cuenoti
Source: BMC Syst Biol. 2013 Oct 31;7:114. doi: 10.1186/1752-0509-7-114 (PMC3819652; doi:10.1186/1752-0509-7-114)
Supplement: Additional file 1 — Schematic representation of UMs. The file contains schemes and a brief description of each UM. [file 1752-0509-7-114-S1.pdf]

Supplementary Material 1.

Figures representing the structure of each UM after the complete curation of the model, for the case where it was possible to solve the inconsistencies. The UMs composed by reactions and metabolites that were removed from the previous version of the model [1] are also shown. The rectangles colored with green represent the new activities (or in-fluxes) added to the model that were needed to solve the inconsistencies found in some UMs. Dotted arrows are used to represent the drain of cofactor metabolites into the biomass equation, as well as the import of metabolites from an external source. Finally, the reactions with no gene association (i.e. orphan reactions) are represented by rounded rectangles and highlighted in yellow, while wrong EC Number assignations are represented with orange hexagonal boxes.

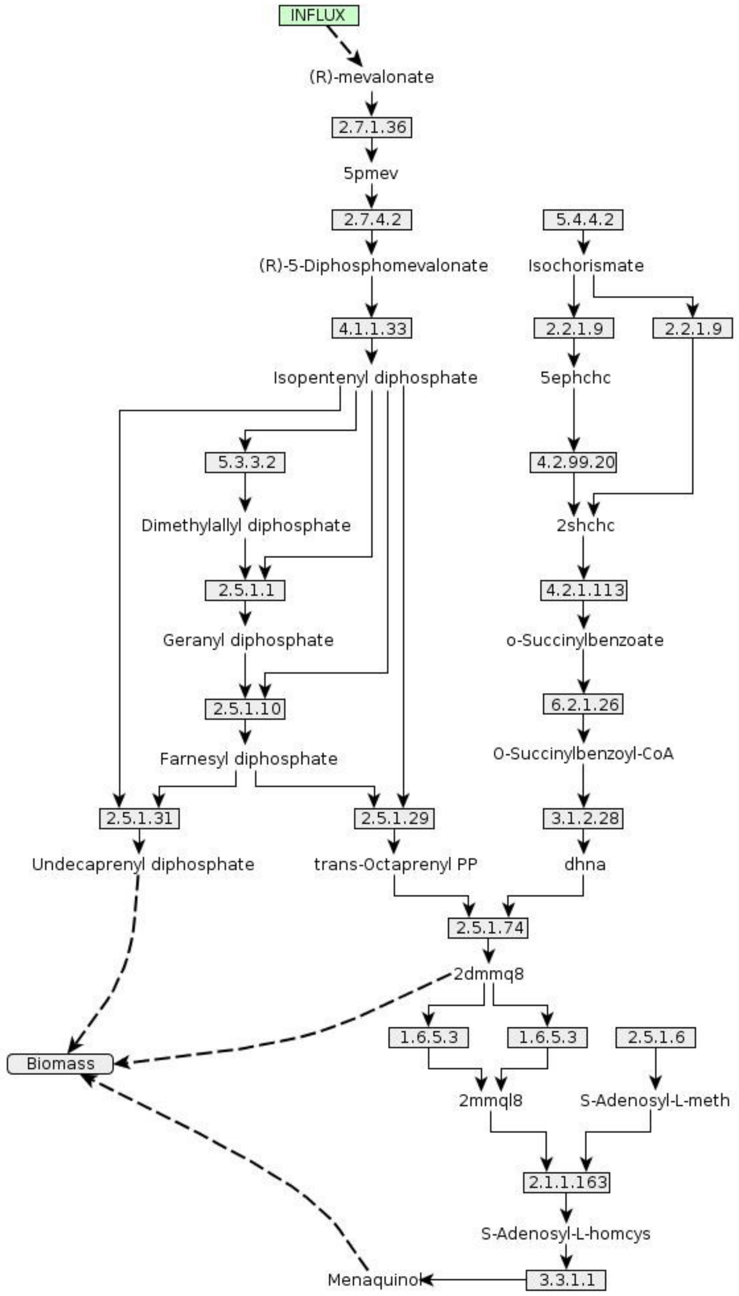

Figure 1. *Schematic representation of UM1 after the modifications introduced to the model in order to solve this inconsistency.*

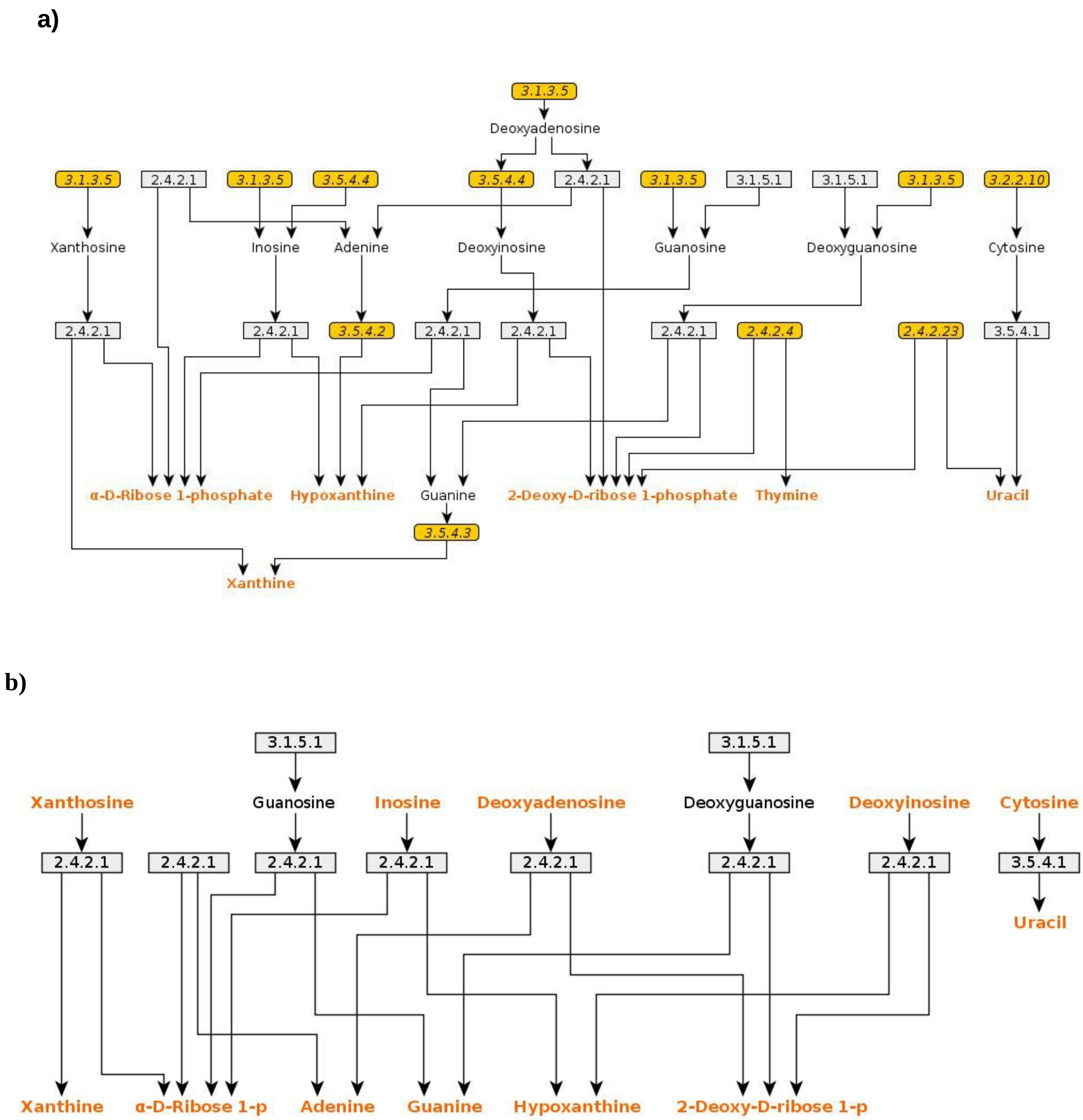

Figure 2. *Schematic representation of UM2 that corresponds with Nucleotide Salvage Pathway. a) Structure of the UM including orphan reactions and wrong annotated activities. b) Structure of the UM after removing all the orphan and wrong assigned activities.*

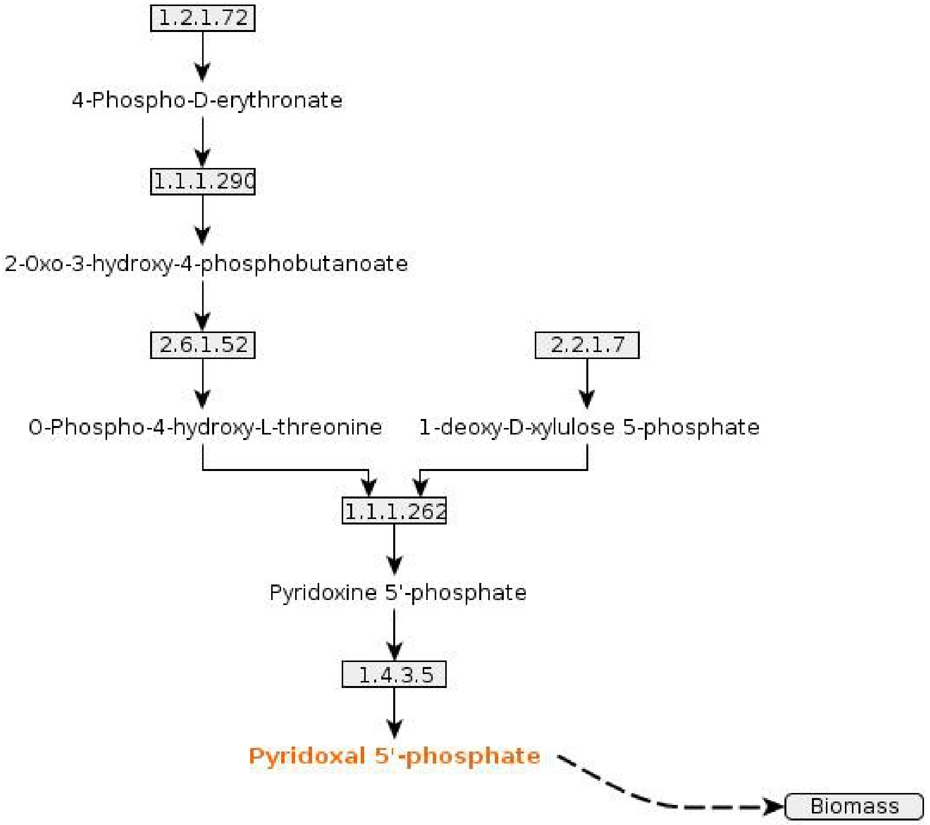

Figure 3. *UM3 represents pyridoxal-5-phosphate biosynthesis with the incorporation of the corresponding cofactor into the biomass equation.*

a)

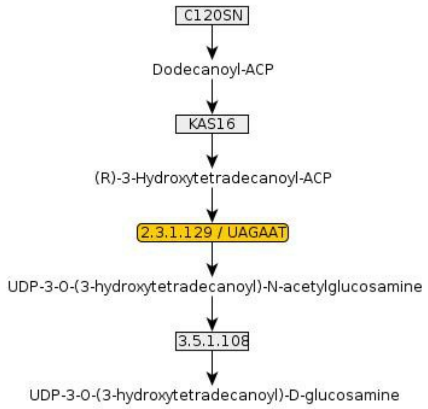
C120SN
Dodecanoyl-ACP
KAS16
(R)-3-Hydroxytetradecanoyl-ACP
2.3.1.129 / UAGAAT
UDP-3-O-(3-hydroxytetradecanoyl)-N-acetylglucosamine
3.5.1.108
UDP-3-O-(3-hydroxytetradecanoyl)-D-glucosamine

b)

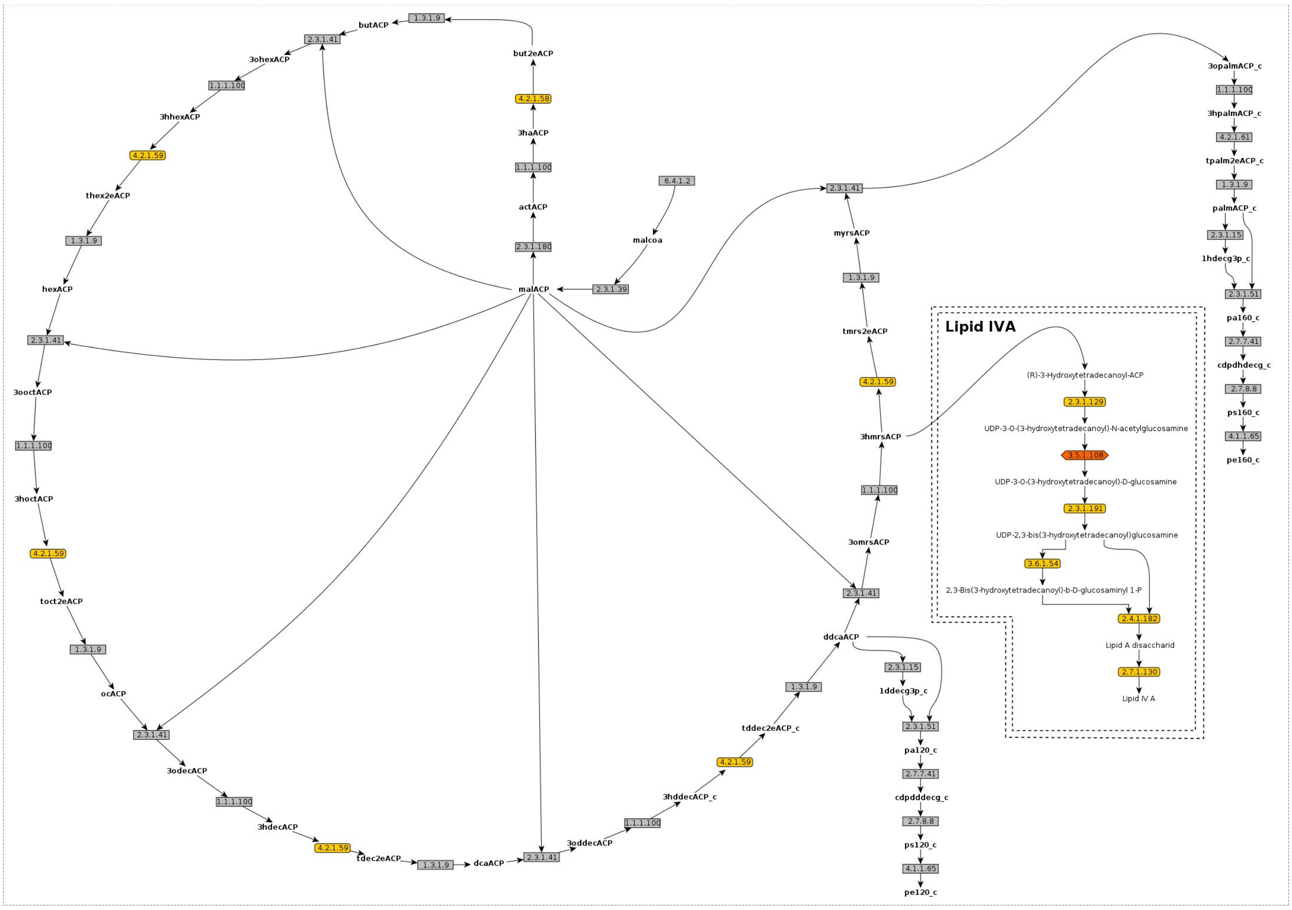
Lipid IVA
(R)-3-Hydroxytetradecanoyl-ACP
2.3.1.129
UDP-3-O-(3-hydroxytetradecanoyl)-N-acetylglucosamine
3.5.1.108
UDP-3-O-(3-hydroxytetradecanoyl)-D-glucosamine
2.3.1.191
UDP-2,3-bis(3-hydroxytetradecanoyl)glucosamine
3.6.1.54
2,3-Bis(3-hydroxytetradecanoyl)-b-D-glucosaminyl 1-P
2.4.1.182
Lipid A disaccharid
2.7.1.130
Lipid IV A
malACP
malcoa
6.4.1.2
2.3.1.39
2.3.1.180
actACP
1.1.1.100
3haACP
4.2.1.58
but2eACP
1.3.1.9
butACP
2.3.1.41
3ohexACP
3hhexACP
4.2.1.59
thex2eACP
hexACP
3ooctACP
3hoctACP
toct2eACP
ocACP
3odecACP
3hdecACP
tdec2eACP
dcaACP
3oddecACP
3hddecACP_c
tddec2eACP_c
ddcaACP
3omrsACP
3hmrsACP
tmrs2eACP
myrsACP
3opalmACP_c
3hpalmACP_c
4.2.1.61
tpalm2eACP_c
palmACP_c
2.3.1.15
1hdecg3p_c
2.3.1.51
pa160_c
2.7.7.41
cdpdhdecg_c
2.7.8.8
ps160_c
4.1.1.65
pe160_c
1ddecg3p_c
pa120_c
cdpdddecg_c
ps120_c
pe120_c

c)

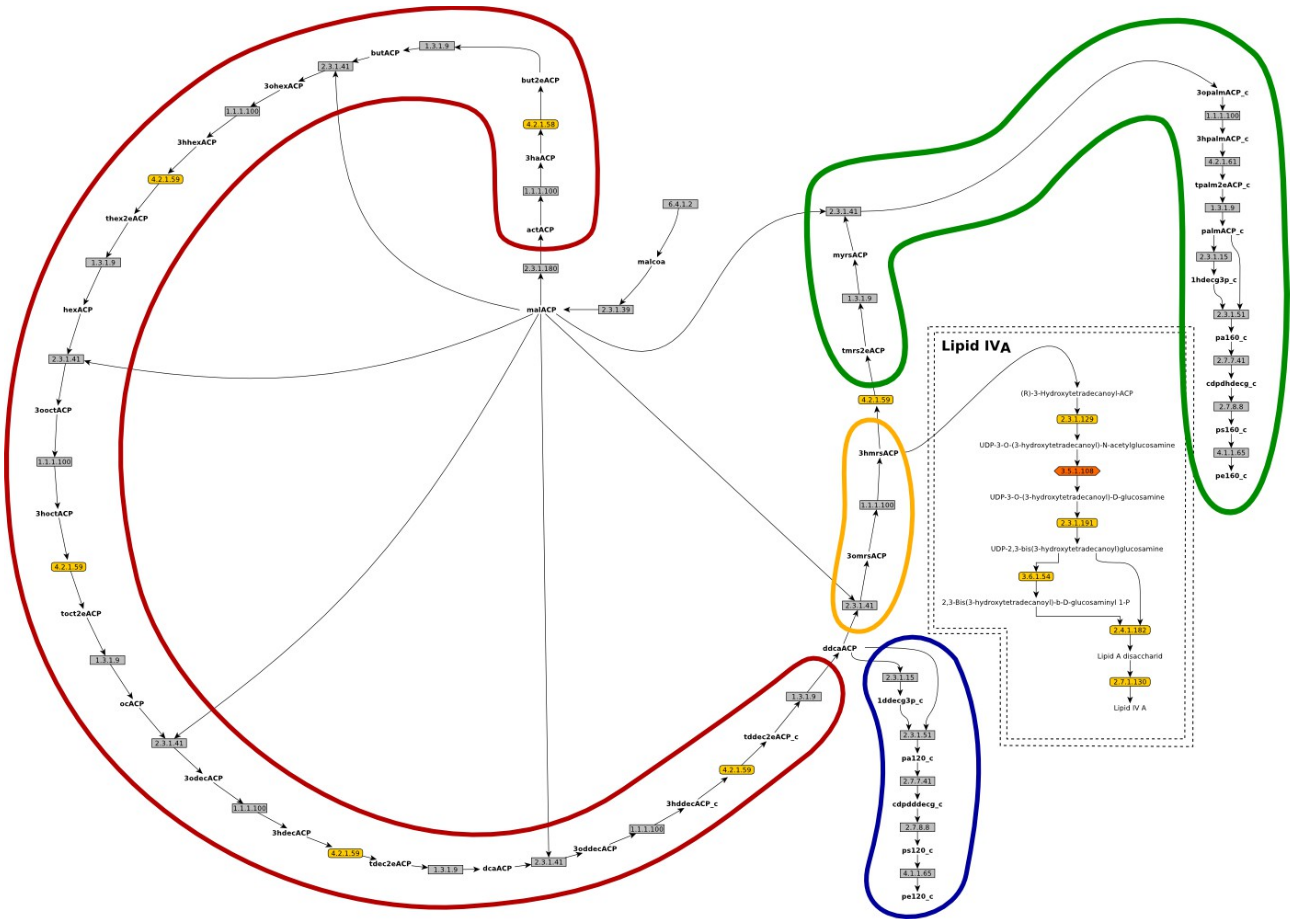

***Figure 4**. Different representations of UM4, which is related to the biosynthetic pathways of different membrane lipids, are shown. Part **a)** represents the UM4 as it is found in the model iCG238. In this case it is worth to note that reaction C120SN is the net sum of 19 activities that produce dodecanoyl-ACP from acetoacetyl-ACP. The reaction KAS16 is also the condensation of the activities 2.3.1.41 and 1.1.1.100. Part **b)** represents a reformulated version of UM4, where reactions C120SN and KAS16 have been "decondensed". This graph also includes the branch pathways that produce two different phosphatidylethanolamine species and the lipid IVA. In this representation, it is easy to see that the activities that participate in the lipid IVA biosynthetic pathway are all orphan except for the activity 3.5.1.108 (orange box), which has been assigned to the gene BLBBGE_037 [GenBank: CP001487]. This scenario suggests a possible error in the annotation of BLBBGE_037 [GenBank: CP001487]. Indeed, it was found that this gene has an ortholog in other Blattabacterium strains and these orthologs have been annotated as the coding gene for activities 4.2.1.58 and 4.2.1.59, which in term seems to be orphan in iCG238. The resolution of this UM means to change the assigned activity from 3.5.1.108 to 4.2.1.58 and 4.2.1.59 in gene BLBBGE_037 [GenBank: CP001487]. **c)** Reactions of part b) have been roughly grouped as appear described in model iCG238: red C120SN ; red + blue + yellow + green = PASYN_Bge DASYN_Bge + PSSA_Bge + PSD_Bge +C140SN + C141SN + C160SN + C161SN + C181SN.*

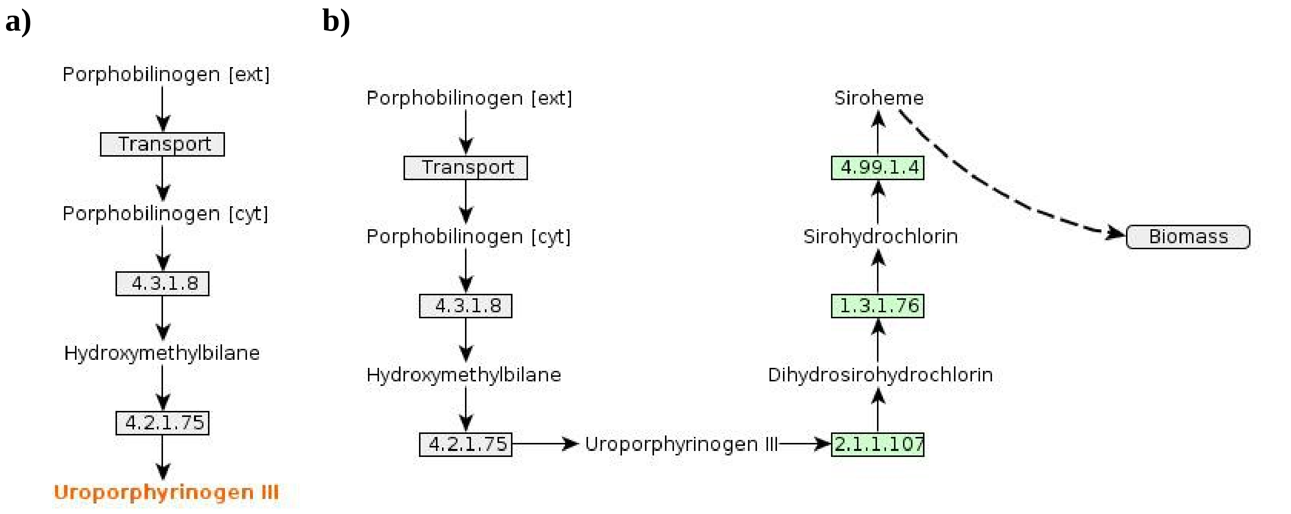

Figure 5. *Representation of UM5 corresponding to siroheme biosynthetic pathway.* ***a)*** *Partial structure of the pathway as it is found in iCG238.* ***b)*** *Complete pathway recovered after finding two genes, BLBBGE_278 [GenBank: CP001487] and BLBBGE_281 [GenBank: CP001487], coding for three missing activities. In particular it was found that BLBBGE_278 [GenBank: CP001487] codes for activities 2.1.1.107 and 4.99.1.4 whereas BLBBGE_281 [GenBank: CP001487] codes for activity 1.3.1.76. Finally, siroheme was included in the biomass equation.*

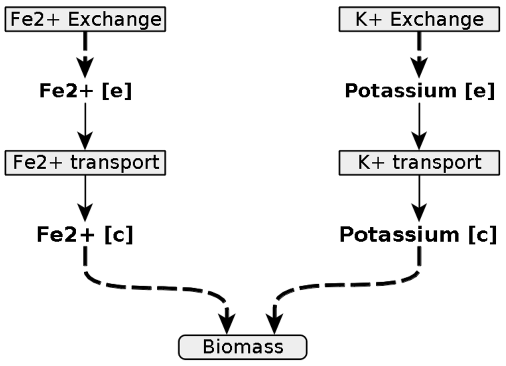

Figure 6. *Representation of UMs 7 and 8. In both cases the corresponding product was included in the biomass equation for solving the UM.*

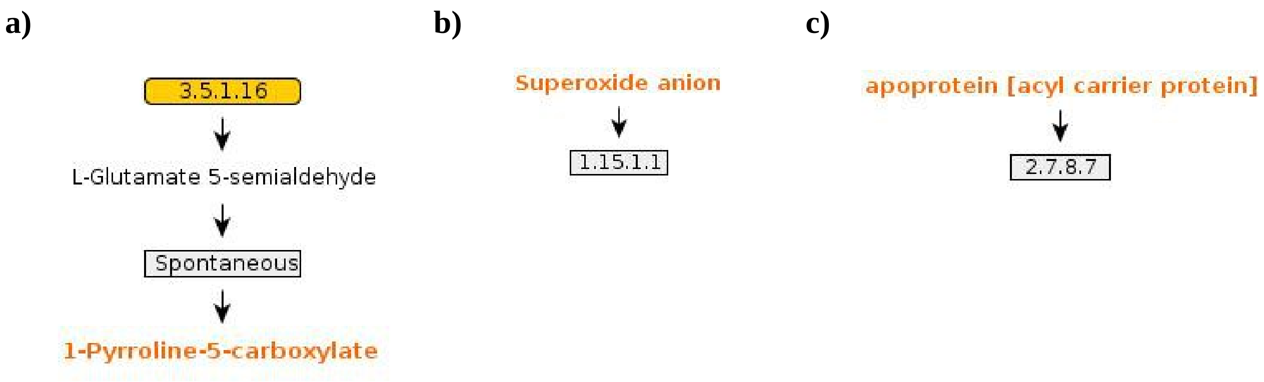

Figure 7. a) *UM6 was found to be a reconstruction error and thus was removed in the new version of the model (iMP242).* ***b)*** *UM 9, composed by the activity 1.15.1.1, was excluded from the model because the*

process of superoxide formation is not considered in the stoichiometric modeling. ***c)*** *UM10 represented by the activity 2.7.8.7, was also excluded from the model because the process of apoprotein synthesis is out of the scope of the model.*

References

1. González-Domenech CM, Belda E, Patiño-Navarrete R, Moya A, Peretó J, Latorre A: **Metabolic stasis in an ancient symbiosis: genome-scale metabolic networks from two Blattabacterium cuenoti strains, primary endosymbionts of cockroaches.** *BMC microbiology* 2012, **12 Suppl 1**:S5.
